# A novel semi-supervised algorithm for the taxonomic assignment of metagenomic reads

**DOI:** 10.1186/s12859-015-0872-x

**Published:** 2016-01-06

**Authors:** Vinh Van Le, Lang Van Tran, Hoai Van Tran

**Affiliations:** Faculty of Computer Science and Engineering, HCMC University of Technology, 268 Ly Thuong Kiet, Q10, HCM City, Vietnam; Institute of Applied Mechanics and Informatics, Vietnam Academy of Science and Technology, 01 Mac Dinh Chi, Q1, HCM City, Vietnam; Faculty of Information Technology, Lac Hong University, 10 Huynh Van Nghe, Bien Hoa, Dong Nai Vietnam; Faculty of Information Technology, HCMC University of Technology and Education, 1 Vo Van Ngan, Thu Duc, HCM City, Vietnam

**Keywords:** Metagenomics, Taxonomic assignment, Semi-supervised learning, DNA sequences, Similarity search

## Abstract

**Background:**

Taxonomic assignment is a crucial step in a metagenomic project which aims to identify the origin of sequences in an environmental sample. Among the existing methods, since composition-based algorithms are not sufficient for classifying short reads, recent algorithms use only the feature of similarity, or similarity-based combined features. However, those algorithms suffer from the computational expense because the task of similarity search is very time-consuming. Besides, the lack of similarity information between reads and reference sequences due to the length of short reads reduces significantly the classification quality.

**Results:**

This paper presents a novel taxonomic assignment algorithm, called SeMeta, which is based on semi-supervised learning to produce a fast and highly accurate classification of short-length reads with sufficient mutual overlap. The proposed algorithm firstly separates reads into clusters using their composition feature. It then labels the clusters with the support of an efficient filtering technique on results of the similarity search between their reads and reference databases. Furthermore, instead of performing the similarity search for all reads in the clusters, SeMeta only does for reads in their subgroups by utilizing the information of sequence overlapping. The experimental results demonstrate that SeMeta outperforms two other similarity-based algorithms on different aspects.

**Conclusions:**

By using a semi-supervised method as well as taking the advantages of various features, the proposed algorithm is able not only to achieve high classification quality, but also to reduce much computational cost. The source codes of the algorithm can be downloaded at http://it.hcmute.edu.vn/bioinfo/metapro/SeMeta.html

**Electronic supplementary material:**

The online version of this article (doi:10.1186/s12859-015-0872-x) contains supplementary material, which is available to authorized users.

## Background

Metagenomics is a powerful approach for studying microbial samples, without the needs of isolating and culturing single organisms. The discipline offers opportunities to discover the complexity and diversity of microbial communities. Earlier metagenomic projects have provided a good understanding of various microbial environments such as acid mine drainage [1], seawater [2], and human gut [3]. With the development of the next-generation sequencing (NGS) techniques, e.g., 454 pyrosequencing, Illumina Genome Analyzer, AB SOLiD [4], current metagenomic projects usually process an unprecedented amount of biological data. Moreover, reads generated by the NGS techniques are often less than 700 bp [5]. For instance, current Illumina read lengths are from 36 to 300 bp (single-end or paired-end reads). These aspects pose major challenges for computational analysis [6, 7].

One of the crucial tasks in a metagenomic project, referred to as *taxonomic assignment* problem, is to identify the origin of each sequence in an environmental sample. This task helps in grouping the sequences into bins and determining how they are related to known taxa. Current taxonomic assignment algorithms are mainly based on the *composition* and *similarity* features of genomic sequences.

Some algorithms only use composition features, e.g., GC-content (TAC-ELM [8]), oligonucleotide frequencies (TACOA [9], MetaID [10], AKE [11]), which are extracted from both analyzed and reference sequences. Most of those algorithms are proposed to process long reads (≥1000 bp), and consequently to be inaccurate in short read classification, though they are really fast. For example, TACOA can only achieve the sensitivity values from 3 *%* to 17 *%* for reads as short as 800 bp at the taxonomic levels of order and genus. It is clear that the lack of composition information in short reads results in the low classification performance of those methods. Besides, some unsupervised binning methods [12–14] use composition features, but they do not assign taxonomic identity for reads.

Recent taxonomic assignment methods are commonly based on the similarity information between analyzed sequences and sequences in reference databases, which can be obtained by an alignment tool (e.g., BLAST, BLAT). MEGAN [15] is a similarity-based method using the lowest common ancestor (LCA) algorithm to find the best common ancestor based on BLAST bit-scores. One of the drawbacks of the LCA is that ambiguous hits may result in assigning reads to taxonomic levels higher than those of their origin. MEGAN overcomes the problem by using some thresholds related to the bit-scores to filter out the ambiguous hits. Other BLAST-based algorithms, SOrt-ITEMS [16], and CARMA3 [17], also attempt to address the drawback by using a reciprocal search step to identify significant hits. The similarity-based methods are demonstrated to be able to work with short reads. However, a large percentage of reads cannot be classified because those reads do not match to reference sequences or match with extremely low bit-scores. Besides, those methods are very time-consuming because the task of similarity search requires an enormous amount of time.

Utilizing the advantages of the combined usage of composition and similarity features are major motivations for currently available hybrid algorithms. In order to reduce computational time, but still retaining the accuracy like similarity-only based methods, SPHINX [18] firstly classifies reference sequences into clusters, and computes the distance between each query sequence and the centriod of the clusters. The algorithm then only needs to perform BLAST search for each query against sequences in a cluster, instead of the whole reference sequences. MetaCluster-TA [19] and PhymmBL [20] are also known as hybrid algorithms. PhymmBL, an extension of Phymm [20], uses BLAST tool to perform similarity search for all reads to provide reference information supporting for the classification process in Phymm. MetaCluster-TA, on the other hand, can be classified as a semi-supervised method, is a combination of three available algorithms, IDBA-UD [21] for assembling reads into virtual contigs, MetaCluster 5.0 [22] for clustering the contigs as well as unassembled sequences, and MEGAN [15] for labeling clusters. The two algorithms aim to improve the classification quality, but this could make them suffer from more computational expense than similarity-only based methods. Moreover, due to the usage of MEGAN, MetaCluster-TA does not combinedly use the similarity information of reads with reference sequences in each cluster in the process of cluster labeling.

In this paper, we present a new taxonomic assignment algorithm which uses a semi-supervised cluster-and-label method for metagenomic reads. The proposed algorithm, called SeMeta (i.e., a *se*mi-unsupervised taxonomic assignment of *meta*genomic reads), aims to improve both the quality and computational efficiency of the classification for short reads which sufficiently overlap each other. After separating reads into clusters, the proposed method assigns each cluster to the best suitable taxon basing on the similarity between their reads and reference databases. Two main new ideas contributed in this work mainly support to the assignment step of the clusters, utilizing output of the clustering process. Firstly, instead of performing the similarity search of all reads in the clusters against reference databases, the method only needs to do for their subgroup of non-overlapping reads so that it can help in reducing overall run-time significantly. Secondly, the similarity information of reads with reference sequences in each cluster are combinedly used for the cluster assignment to produce better classification quality.

The next section presents the details of the proposed algorithm. The strength of SeMeta on both simulated and real metagenomic datasets is demonstrated in the section of experimental results. The final sections are for discussions and conclusions.

## Methods

### Fundamentals of proposed method

Semi-supervised learning has been known as an efficient technique in many fields, especially in the fields of labeling a large amount of data. It is expected that the technique helps more data items to be labeled, and the labeling to be more accurate comparing to supervised algorithms due to the support of unsupervised process. Several semi-supervised classification methods have been proposed in the literature [23]. In this study, we proposed a semi-supervised algorithm which can be classified as a cluster-and-label method [23]. The proposed algorithm is based on an assumption that reads tend to form separated clusters, and reads in the same cluster are more likely to share a same label.

**Fig. 1:**
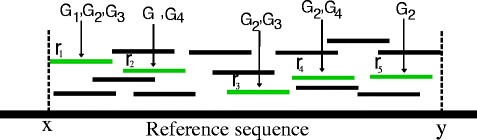
A subset of non-overlapping reads in a cluster. A cluster consists of 16 reads. A subset of 5 non-overlapping reads from *r*_1_ to *r*_5_ covers most of positions from *x* to *y* in the reference sequence. Those reads are also mapped with reference sequences *G*_1_,*G*_2_,*G*_3_,*G*_4_ with high bit-scores

Given a list of *n* metagenomic reads. By using the above assumption, the first step of the proposed method aims to partition the *n* reads into *k* sets *C*_1_,*C*_2_,…,*C*_*k*_,*k*≤*n*. In the second step, each cluster *C*_*i*_ is labeled based on the similarity search of its reads against reference databases. One of the ideas applied in this study is that instead of performing the similarity search for all reads in each cluster, our method only does for its representative defined as follows. Each representative *K*(*C*_*i*_) of a cluster *C*_*i*_,1≤*i*≤*k*, called *a core* of *C*_*i*_, is its subset which contains only non-overlapping reads. This is motivated by an observation that although a core *K*(*C*_*i*_) consists of a small number of reads of *C*_*i*_, it still keeps most of the sequence information of *C*_*i*_. It thus contains the majority of the similarity information between *C*_*i*_ and reference sequences. The idea is exemplified in Fig. 1. Given a cluster consisting of 16 reads which covers from position *x* to *y* in a reference sequence. Choosing a subset of the cluster consisting 5 reads from *r*_1_ to *r*_5_. It can be seen that the subset also covers most of positions from *x* to *y* in the reference sequence. An experiment conducted in this work (presented in the section of Experimental results) demonstrates that the usage of cluster cores has an extremely light effect on the classification quality while reducing much computation cost. Besides, it can be realized that the cores of clusters are similar to assembled contigs which are possibly generated from the reads. The procedure applied in this work helps to avoid an assembly process which is very time-consuming [24] while still keeping classification quality.

In order to determine whether two reads *r*,*s*∈*R* overlap each other or not, this work uses a same method as described in [25] which is based on the number of shared *l*-mers between reads. It is stated as follows. Given $m,l\in N$ (*l* is sufficiently large), if *r*, and *s* share at least *ml*-mers, the two reads are considered as overlapping reads. Otherwise, they are non-overlapping reads [25].

In the step of cluster labeling, a two-level filtering technique is proposed to reduce insignificant hits of the similarity search output (by BLAST tool). The first level filters out the BLAST hits of low bit-scores for each read by using two basic thresholds min-score and top-percent similar to existing studies [15, 16, 26]. The repeat of short sequences between different organisms may cause for the fact that a read may be mapped against reference sequences of different organisms with all high bit-scores, especially with the case of short reads. Thus, it is difficult to distinguish which hits are reliable by only using the first level filtering. By taking the advantage of the clustering process, an additional filtering is applied at the cluster level. Because reads in the same cluster are more likely to have the same taxon, we label reads in the same cluster together. The proposed method only chooses the hits which are mapped by the majority of reads in the core of clusters. For example, assuming that after performing the similarity search for the core consisting of 5 reads (from *r*_1_ to *r*_5_) in Fig. 1, and applying a filtering at the read level, we have 5 lists of hits corresponding to the reads (the list of hits for read *r*_*j*_ is denoted by *h*_*j*_,1≤*j*≤5). *h*_1_={*G*_1_,*G*_2_,*G*_3_}, *h*_2_={*G*_1_,*G*_4_}, *h*_3_={*G*_2_,*G*_3_}, *h*_4_={*G*_2_,*G*_4_}, *h*_5_={*G*_2_}, in which, *G*_1_,*G*_2_,*G*_3_,*G*_4_ are the names of genomes (BLAST hits). If we choose a threshold is 60 *%*, it means that the hits mapped by at least 0.6×5=3 reads in the cluster core will be chosen. Therefore, the hit *G*_2_ is retained, while the others are discarded.

### Algorithms

**Fig. 2:**
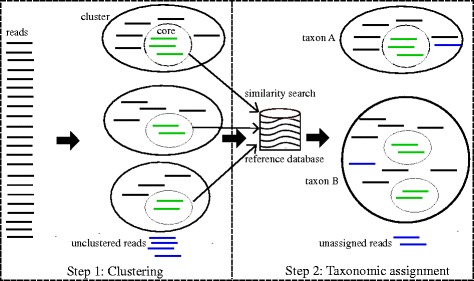
Process of SeMeta. *Step 1* separates reads into clusters, and builds the cluster cores. *Step 2* does similarity searching between the cores and reference sequences, then labels each cluster

This section describes algorithmic aspects of the proposed method in details. Figure 2 presents the process of SeMeta, including two major steps: Clustering and Taxonomic Assignment.

#### Step 1: clustering

In this step, reads are classified into clusters of closely related organisms using an improvement of BiMeta [25] - an efficient clustering algorithm for metagenomic reads. It is similar to BiMeta, the proposed algorithm firstly groups reads based on their overlapping information among them. A *k*-means algorithm is used to merge the groups into clusters basing on extracted *l*-mer frequencies of the groups themselves. However, there are two differences in SeMeta compared to BiMeta. Firstly, since *l*-mer frequencies extracted from extremely small groups are usually not reliable due to the lack of composition information, SeMeta removes them from the phase of group merging to improve the clustering precision. Secondly, while BiMeta requires the number of clusters in data from users, SeMeta is able to detect it automatically by using the evaluation function *f*(*k*) from the study in [27]. The evaluation method has been demonstrated to be effective for *k*-means based algorithms.

#### Building cores of clusters

After reads are separated into *k* clusters *C*_1_,…,*C*_*k*_, cores of the clusters are built based on the information of overlapping sequence between reads. A core *K*(*C*_*i*_),1≤*i*≤*k*, of a cluster *C*_*i*_, which will be a representative of the cluster, is equivalent to an *independent set* or *stable set* on graphs. An independent set defined on a graph is a set of vertices which does not consist any pair of adjacent vertices [28]. In this work, a greedy heuristic algorithm is applied to find a maximal independent set of the cluster.

In practice, datasets may contain reads of extremely low-abundance genomes. These reads are more likely separated into extremely small groups due to the lack of reads overlapping with them. As a result, they could be removed from the clustering step. As an effort to label the reads, the proposed algorithm considers them as clusters and puts them to the taxonomic assignment process. This means that in this case, *C*_*i*_≡*K*(*C*_*i*_).

#### Step 2: taxonomic assignment

This step consists of the three following tasks:

- *Task 1 - Similarity search*: All reads in cluster cores generated in step 1 are mapped against reference databases by the BLAST tool. As denoted above, ${h}_j,j\in N$, is a list of distinct hits returned by the similarity search for a read *r*_*j*_. Each hit *t*∈*h*_*j*_ has a bit-score denoted by *bs*(*t*).
- *Task 2 - Labeling clusters*: The labeling of each cluster *C*_*i*_,1≤*i*≤*k*, is based on the mapping results of the reads in its core *K*(*C*_*i*_) against reference databases, described in Algorithm 1. The idea behind the algorithm is as follows. Given a list *L*={*h*_*j*_,1≤*j*≤|*L*|}, consisting |*L*| lists of hits returned by the similarity search for all reads *r*_*j*_∈*K*(*C*_*i*_), the algorithm performs a filtering technique at two levels: read level and cluster level.
  - *Read level*: Two parameters min-score *s*_*min*_ and top-percent *p*_*top*_ are applied. The threshold min-score *s*_*min*_ is used to discard the hits of extremely low bit-scores. Among the remaining hits of each read, the second threshold top-percent *p*_*top*_ allows to choose the highest bit-score hits of them.
  - *Cluster level*: This level uses a threshold *o*_*max*_ to reduce further unreliable hits. Let *U* be a set of hits, and $U=\cup _{j=1}^{|L|} h_{j}$. We define a function $$ f:U\to N $$ by *f*(*t*)=*the number of the occurrences of a hit t in L*. By using the function, the algorithm only retains the hits which occur in at least *o*_*max*_ percentage of the lists in *L*. If the value of *o*_*max*_ leads to that all list *h*_*j*_∈*L*,1≤*j*≤|*L*|, are empty, it will be decreased by half once.
  Finally, the Lowest Common Ancestor (LCA) algorithm is used to find the lowest common taxon of remaining hits after the filtering. Cluster *C*_*i*_ as well as all reads *r*∈*C*_*i*_ will be labeled by the common taxon.
- *Task 3 - Post processing*: This task is to merge clusters which have the same label into the same cluster. Some clusters may not be labeled because their reads do not match with any reference sequences or match with extremely low bit-scores. Those reads and the reads of clusters assigned at the highest level of the taxonomy tree will be considered as unassigned reads.

#### Databases

The protein RefSeq database (release 69, January 2, 2015), including 51,661 microbial organisms from the NCBI (National Center for Biotechnology Information), is used as a reference database. In order to validate the proposed method in the aspects of assignment for known and unknown species, different variants of the database are created corresponding to two scenarios:

- *Known species*: This scenario simulates the case that reference databases contain sequences of species in queries.
- *Unknown species*: In this case, sequences of query species are absent in reference databases.

#### Performance metrics

The proposed method is evaluated with metrics which are commonly used in literature [17, 29, 30]. They can be defined as follows. Let *N* be the total number of reads, and *A* be the number of assigned reads. Considering at taxonomic level *i*, let *E*_*i*_ be the number of reads assigned to the correct taxa exactly at this level, and *U*_*i*_ be the number of reads assigned to the correct taxa under this level. Two metrics sensitivity and precision (notated by *sensitivity*_*A*_ and *precision*_*A*_, respectively in this work) can be calculated by the following formulations (From now, when we only mention that reads are assigned *at a taxonomic level*, it means that the reads are assigned *exactly at* or *under* the taxonomic level).

$$sensitivity_{A}\, \text{(at level \textit{i})} = \frac{E_{i} + U_{i}}{N}, $$

$$precision_{A}\, \text{(at level \textit{i})}= \frac{E_{i} + U_{i}}{A}. $$

For example, given a read originating from *Mycoplasma fermentans*, when we consider at genus level, an assignment of the read as *Mycolasma* would increase *E*_*i*_, and *Mycoplasma fermentans, Mycoplasma gallisepticum* would increase *U*_*i*_. These values are computed at five taxonomic levels: species, genus, family, order, and class.

Because each of the metrics precision and sensitivity itself does not fully reflect the performance of an assignment algorithm, we use an additional metric named F-measure emphasizing comprehensively on the both metrics. It is defined as in [31].

$$F-measure_{A}=\frac{2}{\frac{1}{precision_{A}}+ \frac{1}{sensitivity_{A}}}. $$

One of the meaningful goals of metagenomic analysis is to discover the DNA sequences belonging to novel organisms whose genomes are not present in reference databases. This can be measured by calculating the total number of reads assigned to the correct taxa exactly at taxonomic levels supported by the evidence. The assignment to the correct taxa under the taxonomic levels would be counted as incorrect assignment [30]. This study applies the measurement for the database scenario of unknown species as follows.

$$sensitivity_{B} = \frac{\sum_{i \in T} E_{i}}{N}, $$

$$precision_{B} = \frac{\sum_{i \in T} E_{i}}{A}, $$

in which, *T*=*the lowest levels of the correct taxa supported by the evidence*. For example, given a read from a species not present in a reference database. Assuming that sequences from the same family with the organism are available, but no sequences from the same genus are present in the reference database, *E*_*i*_ would be counted exactly at family level.

## Results

SeMeta is compared with two well-known similarity-based algorithms on the RefSeq database: MEGAN [15] (version 5.8.6), and SOrt-ITEMS [16]. Two common parameter thresholds, namely minimum bit-score, and the top-percent, of the three algorithms are set equally of 35 and 10 %, respectively. Remaining parameters of the MEGAN and SOrt-ITEMS are set by default. The max-occur threshold *o*_*max*_ of the proposed algorithm is set of 50 % for all tests. In order to perform the similarity search, the BLASTx tool (version 2.2.30) is downloaded from the NCBI website. The tool runs with the fast mode (parameter -task is blastx-fast), and other parameters are set to default values. Besides, the algorithms use the NCBI taxonomy versions reported in Additional file 1.

***Datasets*** Three simulated datasets (described in Additional file 2: Table S1, S2 and S3), named *ds*1,*ds*2 and *ds*3, respectively, are generated using bacterial genomes from the NCBI database. These datasets are created by MetaSim tool [32] following the Illumina error profile with length of 80 bp, and 100 bp, and an error rate of 1 *%*. The number of genomes in the datasets are 5, 10, and 15. Dataset *ds*1 and *ds*3 consists of genomes which are described in [16, 30], respectively.

SeMeta also is used to analyze two real metagenomes. The first dataset is the Acid Mine Drainage (AMD) dataset [1] which consists of 180,713 sequences, downloaded from NCBI trace archive. The second real dataset is the sample MH0051 containing of human gut metagenomic (HGM) data [3]. It consists of 20,309,712 Illumina paired-end reads with the length of 75 bp.

### Validation of SeMeta on simulated datasets

SeMeta is compared with MEGAN and SOrt-ITEMS on the dataset *ds*1, *ds*2 and *ds*3 for two scenarios of reference databases: known species and unknown species. For the first scenario, it can be seen from Table 1 that SeMeta returns much better results than MEGAN and SOrt-ITEMS at species level. While SOrt-ITEMS is unable to detect any organisms at this level, SeMeta achieves from 10.12 % to 29.46 % *sensitivity*_*A*_ higher than those of MEGAN, and from 0.04 % to 27.54 % *precision*_*A*_ higher those of the method for the three datasets. At the higher levels from genus to class, SeMeta and MEGAN outperform SOrtITEMS in both aspects of the *sensitivity*_*A*_ and the *precision*_*A*_. Although MEGAN gets higher *precision*_*A*_ values than SeMeta in the levels for dataset *ds*1 and *ds*2, the proposed method returns better *sensitivity*_*A*_ values than MEGAN for all cases.

**Table 1:** The performance of MEGAN, SOrt-ITEMS and SeMeta on the simulated datasets at different taxonomic levels - The scenario of known species

Method		Species	Genus	Family	Order	Class
		level	level	level	level	level
Dataset *ds*1						
MEGAN	*S* *e* *n*._*A*_	56.9 %	69.13 %	69.47 %	71.34 %	71.79 %
	*P* *r* *e*._*A*_	67.83 %	**82.42 %**	**82.82 %**	**85.06 %**	**85.59 %**
SOrt-ITEMS	*S* *e* *n*._*A*_	N/A	37.83 %	38.91 %	39.87 %	48.84 %
	*P* *r* *e*._*A*_	N/A	45.83 %	47.14 %	48.31 %	59.17 %
SeMeta	*S* *e* *n*._*A*_	**67.02 %**	**72.96 %**	**72.99 %**	**73.27 %**	**75.19 %**
	*P* *r* *e*._*A*_	**67.87 %**	73.89 %	73.92 %	74.21 %	76.15 %
Dataset *ds*2						
MEGAN	*S* *e* *n*._*A*_	46.78 %	74.94 %	75.71 %	75.85 %	77.42 %
	*P* *r* *e*._*A*_	58.29 %	**93.36 %**	**94.33 %**	**94.49 %**	**96.46 %**
SOrt-ITEMS	*S* *e* *n*._*A*_	N/A	58.51 %	60.7 %	60.8 %	71.91 %
	*P* *r* *e*._*A*_	N/A	74.76 %	77.56 %	77.69 %	91.89 %
SeMeta	*S* *e* *n*._*A*_	**76.24 %**	**86.01 %**	**86.09 %**	**86.11 %**	**90.5 %**
	*P* *r* *e*._*A*_	**77.1 %**	86.98 %	87.07 %	87.09 %	91.52 %
Dataset *ds*3						
MEGAN	*S* *e* *n*._*A*_	36.82 %	49.55 %	57.54 %	58.1 %	61.68 %
	*P* *r* *e*._*A*_	42.91 %	57.76 %	67.07 %	67.73 %	71.89 %
SOrt-ITEMS	*S* *e* *n*._*A*_	N/A	34.88 %	50.12 %	55.96 %	63.2 %
	*P* *r* *e*._*A*_	N/A	42.48 %	61.04 %	68.15 %	76.97 %
SeMeta	*S* *e* *n*._*A*_	**61.03 %**	**68.33 %**	**76.57 %**	**77.07 %**	**81.14 %**
	*P* *r* *e*._*A*_	**70.45 %**	**78.88 %**	**88.39 %**	**88.97 %**	**93.67 %**

N/A = Not Available. The bold values indicate the best results among the algorithms in the aspect of *s* *e* *n* *s* *i* *t* *i* *v* *i* *t* *y*_*A*_ (*S* *e* *n*._*A*_) or *p* *r* *e* *c* *i* *s* *i* *o* *n*_*A*_ (*P* *r* *e*._*A*_)

**Fig. 3:**
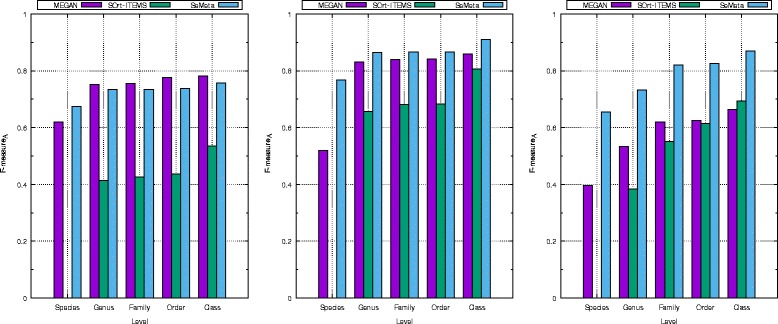
The *F*- *m* *e* *a* *s* *u* *r* *e*_*A*_ of MEGAN, SOrt-ITEMS, and SeMeta on simulated datasets for the scenario of known species. The left chart is for the dataset *d* *s*1, the middle chart is for dataset *d* *s*2, and the right chart is for the dataset *d* *s*3

Figure 3 presents the *F*- *measures*_*A*_ of the three algorithms, which reflects the overall classification quality of them in this scenario. At species level, SeMeta achieves 5.5 %, 24.77 %, 25.77 % *F*- *measure*_*A*_ higher than those of MEGAN for dataset *ds*1, *ds*2 and *ds*3, respectively. At the remaining levels, MEGAN gets slightly higher *F*- *measures*_*A*_ than those of SeMeta for dataset *ds*1. Conversely, SeMeta returns better *F*- *measures*_*A*_ than those of MEGAN for dataset *ds*2 and *ds*3.

Table 2 shows the experimental results of the three algorithms for the scenario of unknown species. Because all sequences of species in each dataset are removed from the reference database (RefSeq database), we only validate the methods at genus level to higher levels. It is interesting that SeMeta also achieves much better *precision*_*A*_ and *sensitivity*_*A*_ than those of MEGAN and SOrt-ITEMS at genus level for the three datasets. SeMeta gets from 5.24 % to 46.91 % *sensitivity*_*A*_, and from 2.82 % to 47.57 % *precision*_*A*_ higher than those of the two remaining methods.

**Table 2:** The performance of MEGAN, SOrt-ITEMS, and SeMeta on the simulated datasets at different taxonomic levels - The scenario of unknown species

Method		Genus	Family	Order	Class
		level	level	level	level
Dataset *ds*1					
MEGAN	*S* *e* *n*._*A*_	50.35 %	51.63 %	59.38 %	60.15 %
	*P* *r* *e*._*A*_	70.36 %	72.14 %	82.98 %	84.05 %
SOrt-ITEMS	*S* *e* *n*._*A*_	21.05 %	27.96 %	31.91 %	41.05 %
	*P* *r* *e*._*A*_	30.11 %	40 %	45.66 %	58.73 %
SeMeta	*S* *e* *n*._*A*_	**67.66 %**	**74.64 %**	**75.38 %**	**76.59 %**
	*P* *r* *e*._*A*_	**77.68 %**	**85.71 %**	**86.57 %**	**87.95 %**
Dataset *ds*2					
MEGAN	*S* *e* *n*._*A*_	56.14 %	58.74 %	59.1 %	61.39 %
	*P* *r* *e*._*A*_	83.23 %	**87.08 %**	**87.62 %**	91.05 %
SOrt-ITEMS	*S* *e* *n*._*A*_	31.54 %	39.69 %	40.05 %	52.6 %
	*P* *r* *e*._*A*_	48.05 %	60.46 %	61.02 %	80.14 %
SeMeta	*S* *e* *n*._*A*_	**78.45 %**	**78.53 %**	**78.55 %**	**83.06 %**
	*P* *r* *e*._*A*_	**86.05 %**	86.13 %	86.15 %	**91.09 %**
Dataset *ds*3					
MEGAN	*S* *e* *n*._*A*_	32.18 %	52.48 %	56.32 %	61.78 %
	*P* *r* *e*._*A*_	41.43 %	67.57 %	72.51 %	79.54 %
SOrt-ITEMS	*S* *e* *n*._*A*_	9.34 %	28.5 %	34.19 %	44.42 %
	*P* *r* *e*._*A*_	12.45 %	37.98 %	45.57 %	59.2 %
SeMeta	*S* *e* *n*._*A*_	**37.41 %**	**58.64 %**	**60.38 %**	**71.46 %**
	*P* *r* *e*._*A*_	**49.37 %**	**77.39 %**	**79.68 %**	**94.31 %**

The bold values indicate the best results among the algorithms in the aspect of *s* *e* *n* *s* *i* *t* *i* *v* *i* *t* *y*_*A*_ (*S* *e* *n*._*A*_) or *p* *r* *e* *c* *i* *s* *i* *o* *n*_*A*_ (*P* *r* *e*._*A*_)

**Fig. 4:**
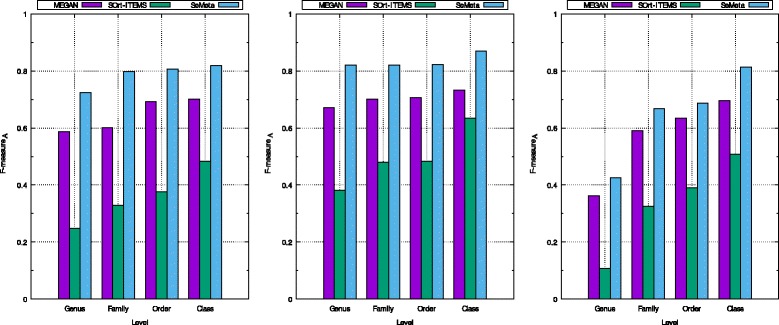
The *F*- *m* *e* *a* *s* *u* *r* *e*_*A*_ of MEGAN, SOrt-ITEMS, and SeMeta on simulated dataset for the scenario of unknown species. The left chart is for the dataset *d* *s*1, the middle chart is for dataset *d* *s*2, and the right chart is for the dataset *d* *s*3

For the higher levels from family to class, it is different from the scenario of known species, SeMeta outperforms the remaining algorithms for all cases. Our method achieves higher both *precision*_*A*_ and *sensitivity*_*A*_ than those of MEGAN and SOrt-ITEMS for most of the cases (7 out of 9 cases). Consequently, the *F*- *measures*_*A*_ of our algorithm are much higher than those of the other methods for all cases in this scenario (presented in Fig. 4).

**Fig. 5:**
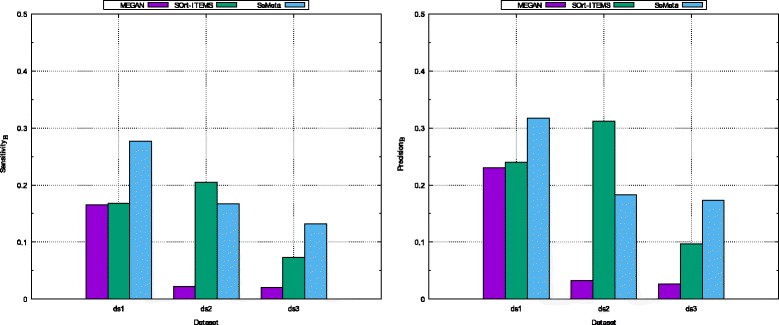
The *s* *e* *n* *s* *i* *t* *i* *v* *i* *t* *y*_*B*_ and *p* *r* *e* *c* *i* *s* *i* *o* *n*_*B*_ of MEGAN, SOrt-ITEMS, and SeMeta in the case of assigning reads exactly at the lowest taxonomic levels supported by the evidence on simulated datasets

In the aspect of assigning reads to the correct taxa exactly at the lowest levels supported by the evidence (for the scenario of unknown species), SeMeta and SOrt-ITEMS get higher *sensitivity*_*B*_ and *precision*_*B*_ than those of MEGAN for the three datasets (Fig. 5). In addition, while SOrt-ITEMS achieves higher results than SeMeta for dataset *ds*2, the proposed algorithm is better than SOrt-ITEMS for dataset *ds*1 and *ds*3.

#### Computational costs

Considering the computational efficiency, we compute the runtime of different steps of SeMeta on dataset *ds*2, and compare them with those of MEGAN, and SOrt-ITEMS. This experiments is conducted on virtual machines with a hardware configuration of 4-core processor, 132 GB RAM, running at 2.4 GHz. It can be seen from the Table 3 that SeMeta spends running time approximately 5.6 times less than those of MEGAN and SOrt-ITEMS (187.67 h compared with 1052.57 h, and 1061 h, respectively). For more details, although SeMeta has to spend time to perform the clustering step while the two other methods do not, the proposed method requires much less runtime than MEGAN and SOrt-ITEMS at the similarity search and assignment steps.

**Table 3:** The running time of MEGAN, SOrt-ITEMS, and SeMeta on dataset *d* *s*2

Methods	Clustering	Similarity search	Assignment	Total	
	runtime (hour)	runtime (hour)	runtime (hour)	(hour)	
MEGAN	N/A	1052	0.57	1052.57	
SOrt-ITEMS	N/A	1052	9	1061	
SeMeta	0.62	187	0.05	187.67	

N/A = Not Available

In addition, the similarity search by BLAST against the reference database (RefSeq) is very time-consuming, and the majority of computational costs of the three algorithms are used for this task. For example, it costs approximately 1052 CPU hours to perform the BLAST search for all 428,674 queries of dataset *ds*2 on our system, and the task accounts for 99.9 % of the total running time of MEGAN on this dataset. Thus, the number of BLAST search against the reference database can help us to estimate roughly the computational costs of the algorithms.

**Fig. 6:**
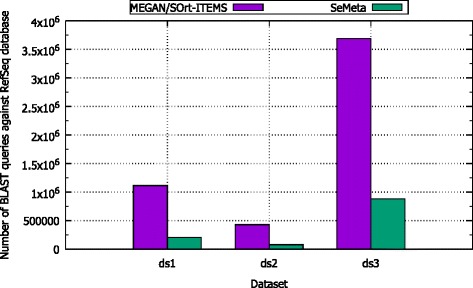
The number of BLAST queries of MEGAN/SOrt-ITEMS, and SeMeta for simulated datasets

Figure 6 presents the number of BLAST queries required by MEGAN or SOrt-ITEMS on three datasets *ds*1, *ds*2, and *ds*3. Since MEGAN and SOrt-ITEMS have to perform the similarity search against reference database for all reads in the datasets, the number of BLAST queries required by the two algorithms is the same (Note that, SOrt-ITEMS also performs the BLAST search at the assignment step, called reciprocal search. However, it does not search against the given reference database, and is not counted in this experiment). Due to the usage of cluster cores, SeMeta requires the number of BLAST queries approximately 4.5 times less than in average those of MEGAN or SOrt-ITEMS for the three datasets.

#### Parameter evaluation

Further experiments are conducted for dataset *ds*2 to validate the impact of parameters on the performance of SeMeta. It can be seen from Additional file 1: Figures S1 to S6 that when parameter min-score *m*_*min*_, and top-percent *p*_*max*_ are not too high (*m*_*min*_≤50,*p*_*max*_≤20 *%*), the classification quality of SeMeta is relatively stable at considered taxonomic levels. In the other hand, the various values of parameter max-occur *o*_*max*_ do not highly affect the performance of SeMeta at class level. However, the proposed algorithm gets high *sensitivity*_*A*_ and *precision*_*A*_ at species level or genus level when 40 *%*≤*o*_*max*_≤60 *%*.

In another aspect, SeMeta achieves high *sensitivity*_*B*_ and *precision*_*B*_ values when 40≤*m*_*min*_≤60 in the case of assigning reads from unknown species exactly at the lowest taxonomic levels supported by the evidence (presented in Additional file 1: Figures S7 to S9). When parameter *p*_*max*_ increases, the trend is that the *sensitivity*_*B*_ and *precision*_*B*_ of SeMeta decrease. In addition, the increase of parameter *o*_*max*_ can increase the *sensitivity*_*B*_, but decrease the *precision*_*B*_ of SeMeta.

#### The effect of the usage of cluster cores

In order to validate the effect of usage of cluster cores on the classification quality, we compare SeMeta with its variant in which all reads in each cluster are used for labeling the cluster, instead of using cluster cores. Additional file 1: Figure S10, S11 and S12 compare the performance of SeMeta with that of the not using core algorithm on dataset *ds*2. It can be seen from the figures that the usage of cluster cores does not much reduce the classification quality. At most of the considered taxonomic levels, SeMeta gets from 0.1 *%* to 1.8 *%* lower *sensitivity*_*A*_ and *precision*_*A*_ than those of the variant algorithm. Besides, not using core algorithm obviously gets slightly better *sensitivity*_*B*_ and *precision*_*B*_ than SeMeta in the aspect of assigning exactly at taxonomic levels. However, from the experiment, SeMeta runs approximately 5 times faster than the variant algorithm.

### Results on real datasets

#### The AMD metagenome

**Fig. 7:**
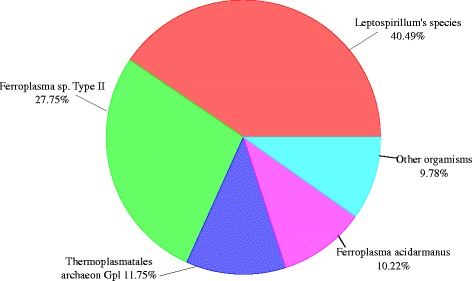
Results of SeMeta on the AMD dataset

By using a traditional method, a study in [1] has revealed that the AMD dataset contains five dominant species: *Leptospirillum sp. Group III, Leptospirillum sp. Group II, Thermoplasmatales archaeon Gpl, Ferroplasma sp. Type II*, and *Ferroplasma acidarmanus*. Among of them, *Ferroplasma sp. Type II*, and *Leptospirillum sp. Group II* have higher abundances than the remaining species. SeMeta is able to assign 79.03 % of the AMD sequences, and returns results (Fig. 7) supporting the previous observation. Our algorithm has detected three species of them including: *Ferroplasma sp. Type II* (27.75 %), *Thermoplasmatales archaeon Gpl* (11.75 %), and *Ferroplasma acidarmanus* (10.22 %). Besides, because the RefSeq database does not contain two species *Leptospirillum sp. Group III* and *Leptospirillum sp. Group II*, SeMeta has detected the existence of genus *Leptospirillum* and some species belonging to the taxon. They account for 40.49 % in the dataset. A small remaining percentage of the dataset belongs to other organisms with 9.78 %. Besides, the number of required BLAST queries accounts for approximately 33.46 % of the number of the AMD sequences.

#### The human gut metagenome

For considering the aspect of the computational efficiency of SeMeta on a large real metagenome, this study conducts an experiment on a sample from the HGM dataset. The experiment shows that SeMeta only performs the similarity search for approximately 20 % of the total number of sequences in the dataset. The list of the most abundant taxa detected by the proposed method is presented in Additional file 2: Table S4 (considering at species level), and Additional file 2: Table S5 (considering at genus level). It can be seen that there are 6 out of the 20 detected species appearing in the list of common microbial species in human gut presented in the study [3], and 11 out of 20 detected genus are in the list. The results demonstrate that SeMeta could be a potential method to work with large metagegomes.

## Discussions

It is a fact that binning algorithms get more difficult to classify reads at lower taxonomic levels. From the experimental results, the proposed method outperforms MEGAN and SOrt-ITEMS at the lowest considered levels (species level for the first scenario, and genus level for the second scenario). This is resulted from the reason that after reads are grouped into clusters during the clustering step, the filtering step by using parameter max-occur *o*_*max*_ at cluster level helps to reduce ambiguous hits successfully, and thus many clusters are assigned correctly to low levels. The technique also performs effectively for the scenario of unknown species. In this case, since all sequences of species present in datasets are removed from reference databases, reads in the datasets are likely to be mapped against reference sequences with low bit-scores, and thus many hits are ambiguous. The filtering makes SeMeta successful in selecting reliable hits, and helps it to classify reads better than the other methods.

In the clustering step of the proposed algorithm, an expected case is that the number of clusters detected automatically is equal or higher than the number of species in datasets. When reads from the same species are separated into different clusters, they are likely to be assigned into the same taxon in the second step of the method. In the case that reads are separated into a smaller number of clusters than the expected one, some clusters which contain reads from different species may be assigned to taxa of higher taxonomic levels (e.g., genus, family, or higher levels).

The prediction of correct taxa for reads from unknown species exactly at the taxonomic levels supported by the evidence, which helps to discover novel organisms directly, is still a challenge. In this aspect, the *sensitivity*_*B*_ and *precision*_*B*_ of the tested methods in the experiments are lower than 38 % for the lowest supported levels. Note that, the results corroborate with the previous experiments [16, 19] in which the number of reads assigned correctly is not high for low taxonomic levels. Thus, this could be a future research direction for our work to improve the classification quality of the proposed algorithm.

## Conclusions

In this paper, we present a semi-supervised method to solve the taxonomic assignment of metagnomic reads. With the support of an unsupervised learning process and an efficient filtering technique at cluster level, the proposed algorithm is able to achieve high classification quality in different aspects. In case of classifying short reads which have sufficient mutual coverage, SeMeta outperforms the two other similarity-based methods. In addition, the usage of reads in cluster cores instead of clusters helps reducing computational costs significantly. For the demand of processing a huge amount of sequences from microbial communities, the algorithm can be used as a promising tool to analyze metagenomic sequences.

## Additional files

Additional file 1
**This file contains the NCBI taxonomy versions used in the experiments, the experimental results to validate the impact of parameters on the classification performance of SeMeta, and the effect of the usage of cluster cores on SeMeta.** (PDF 423 kb)

Additional file 2
**This file contains the details of the simulated datasets used in this study, and the experimental results of SeMeta on a sample of Human Gut Metagenome.** (PDF 90 kb)
