# Supplementary material for: A novel semi-supervised algorithm for the taxonomic assignment of metagenomic reads
Source: BMC Bioinformatics. 2016 Jan 6;17:22. doi: 10.1186/s12859-015-0872-x (PMC4702387; doi:10.1186/s12859-015-0872-x)
Supplement: Additional file 1 — This file contains the NCBI taxonomy versions used in the experiments, the experimental results to validate the impact of parameters on the classification performance of SeMeta, and the effect of the usage of cluster cores on SeMeta. (PDF 423 kb) [file 12859_2015_872_MOESM1_ESM.pdf]

Supplementary Material 1

1 The NCBI taxonomy versions used in experiments

+ SeMeta uses the NCBI taxonomy downloaded on March 27th, 2015 from page: ftp://ftp.ncbi.nih.gov/pub/taxonomy.
+ MEGAN (downloaded on February 13th, 2015) uses the NCBI taxonomy downloaded on November 15, 2014 from the NCBI ftp (from release notes of MEGAN)
+ SOrt-ITEMS (downloaded on November 29th, 2014) uses the NCBI taxonomy updated in June, 2014 (by personal communication with the authors of SOrt-ITEMS).

2 The impact of parameters on the classification performance of SeMeta

This section presents the impact of parameters min-score s_{min}, top-percent p_{top}, and max-occur o_{max} on the classification performance of SeMeta. Those experiments are conducted on dataset $ds2$.

2.1 Parameter min-score s_{min}

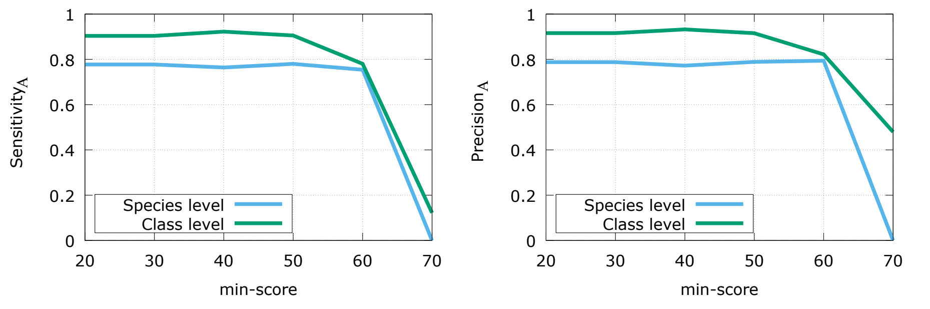

Figure S1 The $sensitivity_A$ and $precision_A$ of SeMeta with different values of parameter min-score s_{min} for the scenario of known species.

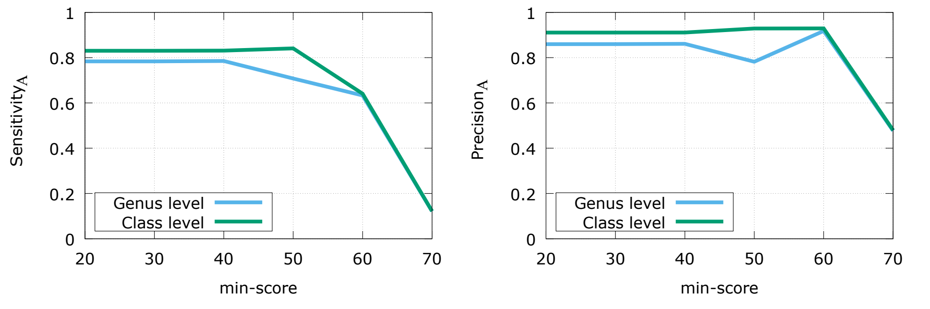

Figure S2 The $sensitivity_A$ and $precision_A$ of SeMeta with different values of parameter min-score s_{min} for the scenario of unknown species.

2.2 Parameter top-percent p_{top}

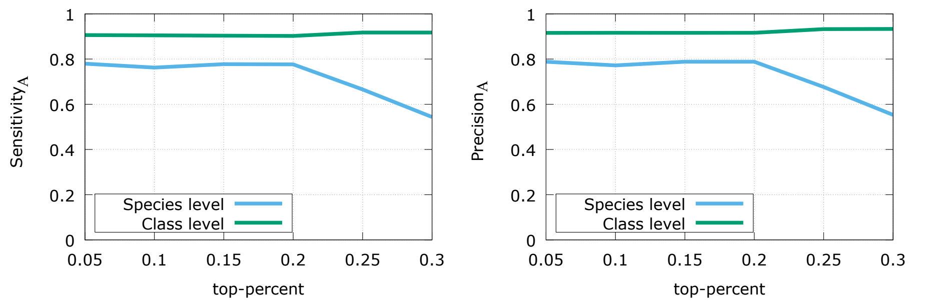

Figure S3 **The** $sensitivity_A$ **and** $precision_A$ **of SeMeta with different values of parameter top-percent** p_{top} **for the scenario of known species.**

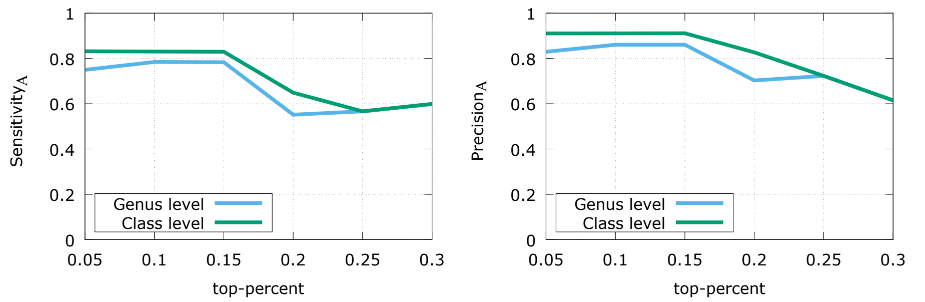

Figure S4 **The** $sensitivity_A$ **and** $precision_A$ **of SeMeta with different values of parameter top-percent** p_{top} **for the scenario of unknown species.**

2.3 Parameter max-occur o_{max}

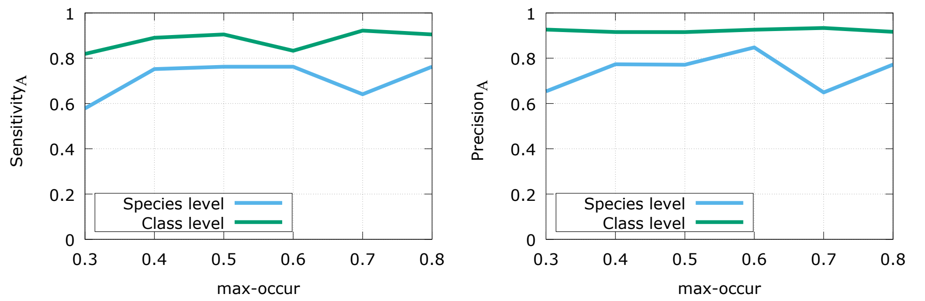

Figure S5 **The** $sensitivity_A$ **and** $precision_A$ **of SeMeta with different values of parameter max-occur** o_{max} **for the scenario of known species.**

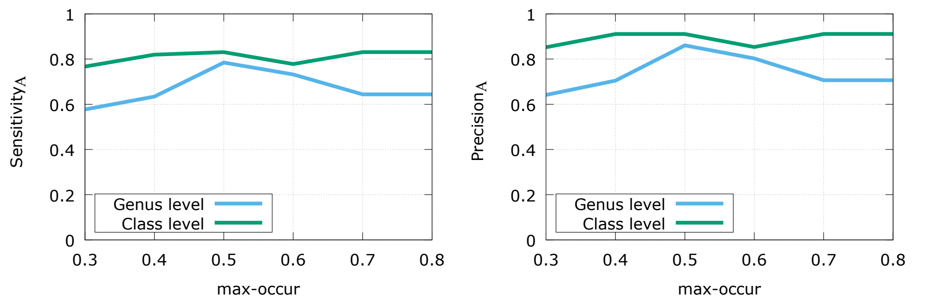

Figure S6 The $sensitivity_A$ and $precision_A$ of SeMeta with different values of parameter max-occur o_{max} for the scenario of unknown species.

2.4 The impact of parameters on SeMeta in the aspect of assigning to correct taxa exactly at the lowest levels

2.4.1 *Parameter min-score* s_{min}

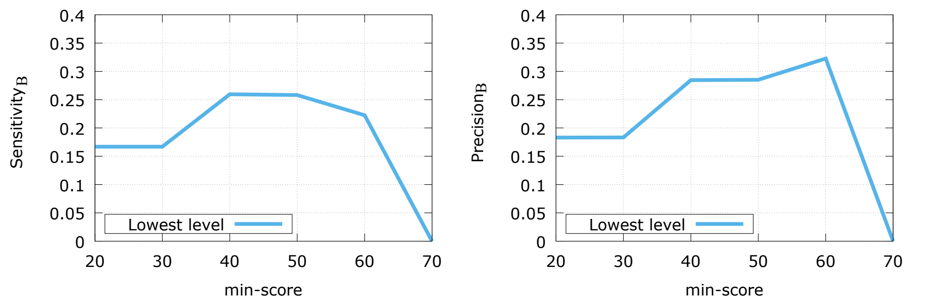

Figure S7 The $sensitivity_B$ and $precision_B$ of SeMeta (assigning exactly at the lowest levels) with different values of parameter min-score s_{min} for the scenario of unknown species.

2.4.2 *Parameter top-percent* p_{top}

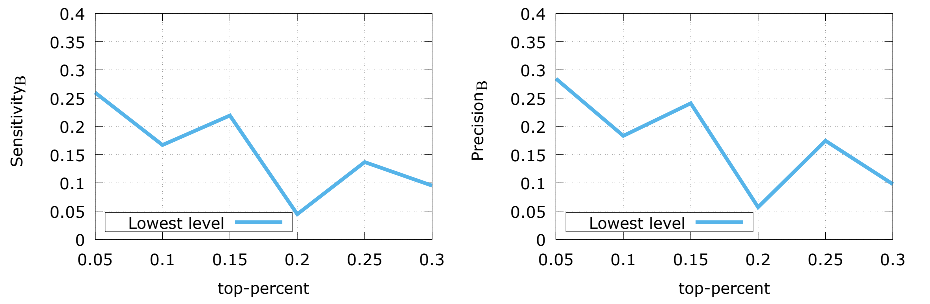

Figure S8 The $sensitivity_B$ and $precision_B$ of SeMeta (assigning exactly at the lowest levels) with different values of parameter top-percent p_{top} for the scenario of unknown species.

2.4.3 *Parameter max-occur* o_{max}

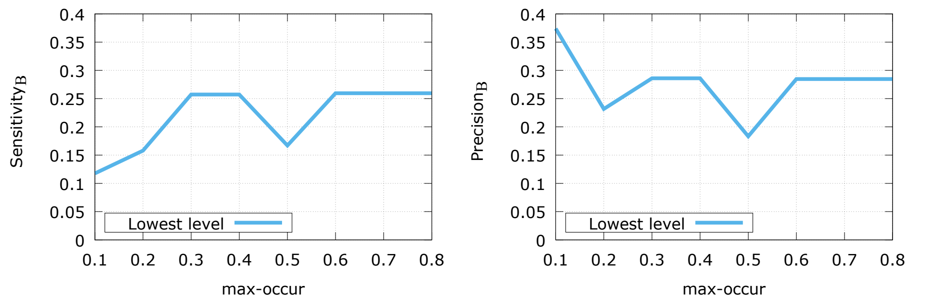

Figure S9 The $sensitivity_B$ and $precision_B$ of SeMeta (assigning exactly at the lowest levels) with different values of parameter max-occur o_{max} for the scenario of unknown species.

3 The effect of the usage of cluster cores on SeMeta

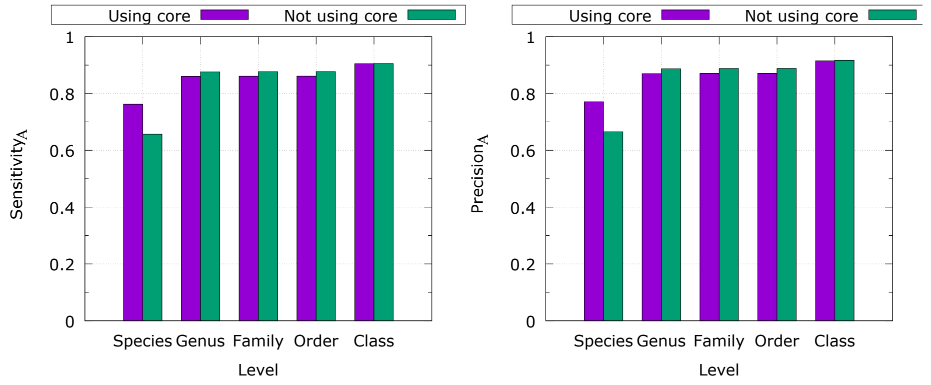

Figure S10 The $sensitivity_A$ and $precision_A$ of SeMeta and its variant which does not use cluster cores on dataset $ds2$, the scenario of known species

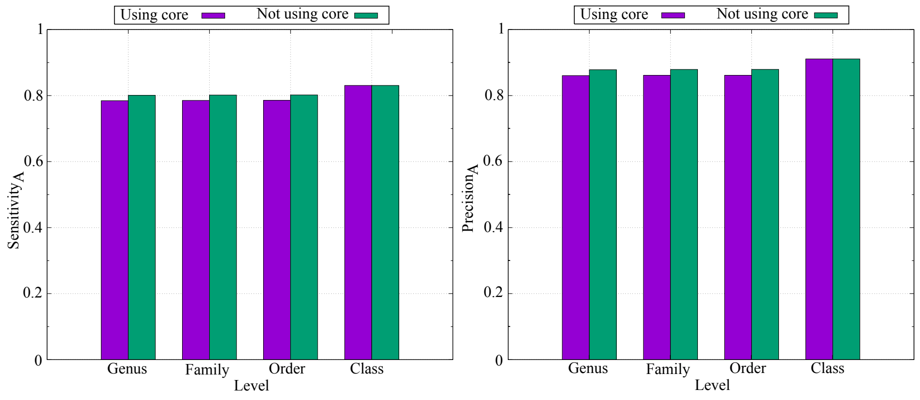

Figure S11 The $sensitivity_A$ and $precision_A$ of SeMeta and its variant which does not use cluster cores on dataset $ds2$, the scenario of unknown species.

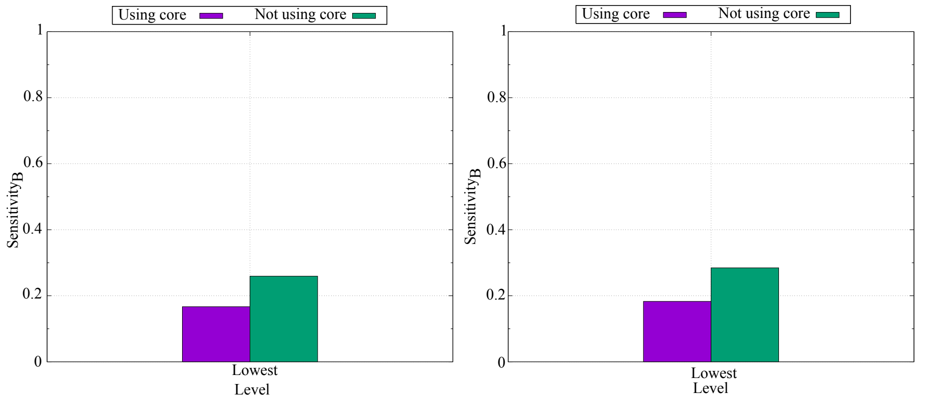

Figure S12 **The $sensitivity_B$ and $precision_B$ of SeMeta and its variant which does not use cluster cores on dataset $ds2$, the scenario of unknown species, and for the aspect of assigning exactly at lowest levels**
