# Supplementary material for: A novel semi-supervised algorithm for the taxonomic assignment of metagenomic reads
Source: BMC Bioinformatics. 2016 Jan 6;17:22. doi: 10.1186/s12859-015-0872-x (PMC4702387; doi:10.1186/s12859-015-0872-x)
Supplement: Additional file 2 — This file contains the details of the simulated datasets used in this study, and the experimental results of SeMeta on a sample of Human Gut Metagenome. (PDF 90 kb) [file 12859_2015_872_MOESM2_ESM.pdf]

Supplementary Material 2

1 The details of simulated datasets

Table S1 Dataset *ds*1

No.	Taxon ID	Species/Strain	Coverage	No. of reads
1	702459	*Bifidobacterium bifidum PRL2010*	5	138042
2	272559	*Bacteroides fragilis NCTC 9343*	5	325808
3	272563	*Clostridium difficile 630*	5	267894
4	226185	*Enterococcus faecalis V583*	5	201704
5	46170	*Staphylococcus aureus subsp. aureus USA300 FPR3757*	5	179106
			Total of reads	1112554

Table S2 Dataset *ds*2

No.	Taxon ID	Species/Strain	Coverage	No. of reads
1	203907	*Candidatus Blochmannia floridanus*	5	44128
2	243230	*Deinococcus radiodurans R1*	5	26208
3	355276	*Leptospira borgpetersenii serovar Hardjo-bovis L550*	5	19826
4	882944	*Listonella anguillarum M3*	5	61452
5	347257	*Mycoplasma agalactiae PG2*	5	54552
6	228908	*Nanoarchaeum equitans Kin4-M*	5	30564
7	222891	*Neorickettsia sennetsu str. Miyayama*	5	53718
8	446468	*Nocardiopsis dassonvillei subsp. dassonvillei DSM 43111*	5	48594
9	262768	*Onion yellows phytoplasma OY-M*	5	53162
10	246198	*Prevotella intermedia 17*	5	36470
			Total of reads	428674

Table S3 Dataset *ds*3

No.	Taxon ID	Species/Strain	Coverage	No. of reads
hline				
1	416269	*Actinobacillus pleuropneumoniae serovar 5b str. L20*	1	22666
2	295405	*Bacteroides fragilis YCH46*	2	105936
3	283166	*Bartonella henselae str. Houston-1*	3	58030
4	568707	*Bordetella bronchiseptica 253*	4	211486
5	224326	*Borrelia burgdorferi B31*	5	44948
6	9	*Buchnera aphidicola*	6	26642
7	243160	*Burkholderia mallei ATCC 23344*	7	245760
8	498211	*Cellvibrio japonicus Ueda107*	8	365770
9	243161	*Chlamydia muridarum Nigg*	9	96928
10	264202	*Chlamydophila felis Fe/C-56*	10	117180
11	243365	*Chromobacterium violaceum ATCC 12472*	11	521158
12	777	*Coxiella burnetii CbuGQ212*	12	241798
13	138119	*Desulfitobacterium hafniense Y51*	13	745622
14	269484	*Ehrlichia canis str. Jake*	14	184028
15	316385	*Escherichia coli str. K-12 substr. DH10B*	15	700742
			Total of reads	3688694

2 The results of SeMeta on a Human Gut dataset

This dataset is the sample MH0051 downloaded from the site: http://public.genomics.org.cn/BGI/gutmeta/. The reference database is RefSeq from the NCBI.

Table S4 List of the most abundant species detected by SeMeta

No.	Species	No. of reads	Is in the list of common taxa of HGM (Qin *et al*, 2010)
1	*Eubacterium barkeri*	2603496	
2	*Pseudomonas fluorescens*	37358	
3	*Faecalibacterium prausnitzii*	26068	×
4	*Pseudomonas aeruginosa*	21096	
5	*Alistipes sp. CAG:268*	11700	
6	*Comamonas testosteroni*	11674	
7	*Firmicutes bacterium*	42642	
8	*Subdoligranulum variabile*	9294	×
9	*Clostridium sp. CAG:288*	8728	
10	*Bacteroides fragilis*	8638	×
11	*Delftia acidovorans*	7622	
12	*Roseburia intestinalis*	7586	×
13	*Streptacidiphilus albus*	7430	
14	*Eubacterium sp. CAG:76*	7280	
15	*Escherichia coli*	6930	×
16	*Anaerotruncus sp. CAG:390*	6288	
17	*Alistipes putredinis*	6036	×
18	*Alistipes timonensis*	5900	
19	*Clostridium sp. CAG:127*	5538	
20	*Burkholderia cepacia*	5518	

Table S5 List of the most abundant genus detected by SeMeta

No.	Genus	No. of reads	Is in the list of common taxa of HGM (Qin *et al*, 2010)
1	*Clostridium*	6575204	×
2	*Eubacterium*	2641976	×
3	*Bacteroides*	1855104	×
4	*Prevotella*	88218	×
5	*Firmicutes*	136950	
6	*Clostridiales*	65760	
7	*Pseudomonas*	62130	
8	*Alistipes*	46386	×
9	*Faecalibacterium*	29700	×
10	*Comamonas*	11894	
11	*Subdoligranulum*	11140	×
12	*Delftia*	7642	
13	*Roseburia*	23118	×
14	*Streptacidiphilus*	7478	
15	*Escherichia*	7036	×
16	*Anaerotruncus*	7550	×
17	*Burkholderia*	15894	
18	*Ruminococcus*	21488	×
19	*Oscillibacter*	11648	
20	*Streptomyces*	9532	
